# Real-World Use of a Mental Health AI Companion: Multiple Methods Study

**DOI:** 10.2196/86904

**Published:** 2026-02-13

**Authors:** Christine Callahan, Leah Tanner, Chelsea Coe, Michelle Davis, Jenna Glover, Ellis Bernstein, Katherine Scranton, Kenli Urruty, Matthew Chester, Sarah Kunkle

**Affiliations:** 1 Headspace, Inc. San Francisco, CA United States; 2 Panasonic Well Austin, TX United States

**Keywords:** mental health, AI, artificial intelligence, digital mental health, Headspace, mental health chatbot

## Abstract

**Background:**

The rapid acceleration of large language models (LLMs) creates opportunities to expand the accessibility of mental health support; however, general artificial intelligence (AI) tools lack safety guardrails, evidence-based practices, and medical regulation compliance, which may result in misinformation and failing to escalate care in crises. In contrast, Ebb, Headspace’s conversational AI tool (CAI tool), was purpose-built by clinical psychologists and research experts using motivational interviewing techniques for subclinical guidance, incorporating clinically backed safety mechanisms.

**Objective:**

This study aimed to (1) understand Headspace members’ sentiment toward AI and expectations for a mental health CAI tool, (2) evaluate real-world use of Headspace’s CAI tool, and (3) understand how members perceive a CAI tool fitting into their mental health journey.

**Methods:**

This was a multiple method study using three data sources including Headspace members: (1) cross-sectional survey (n=482) assessing demographics, AI use, and the Artificial Intelligence Attitude Scale-4 (AIAS-4); (2) real-world engagement descriptive analysis (n=393,969) assessing session and message counts, retention, and conversation themes; and (3) diary study (n=15) exploring the CAI tool’s role within members’ mental health journey. App engagement was compared between CAI tool 1.0 and CAI tool 2.0, where CAI tool 2.0 featured enhanced LLM conversational prompts, comprehensive memory, woven content recommendations, and more robust safety detection.

**Results:**

While the majority of survey respondents used and would continue to use general AI tools, overall attitudes toward AI remained neutral (AIAS-4 mean 5.7, SD 2.2, range 1-10). Survey results suggest that members viewed the CAI tool as a guide to navigate to mental health resources and Headspace content and provide in-the-moment support. Members emphasized the need for data safety and ethics transparency, clinical guidelines structure, and for the CAI tool to be a resource in addition to human-delivered mental health care, not a replacement. Real-world CAI tool use showed strong engagement across 393,969 Headspace members. The product evolution to CAI tool 2.0 led to increased retention (77,894/153,249, 50.8% completed 2 sessions within 7 days vs 68,701/240,720, 28.5% for CAI tool 1.0) and higher positive conversation ratings (37,819/40,449, 93.5% vs 94,308/104,323, 90.4%). Retained CAI tool 2.0 users showed greater retention (6.1 sessions per user) compared to all CAI tool 2.0 users (2.9 sessions per user) and CAI tool 1.0 (2.4 sessions per user). Diary study results suggest that members imagined using the CAI tool when feeling stress or anxiety and during morning routines, commutes, or while winding down at night.

**Conclusions:**

Results emphasize the necessity of research-backed, purpose-built mental health AI products with minimum viable safeguards, including (1) transparent labeling of intended use, benefits, and limitations; (2) safety by design principles to monitor for overuse, detect risk, and flag needs for escalation; and (3) child and adolescent safeguards.

## Introduction

The recent boom in artificial intelligence (AI), accelerated by advancements in large language models (LLMs), has opened new opportunities in mental health as AI-powered tools are being developed to expand access to and accessibility of mental health support. Research suggests that 48.7% of individuals used a general LLM, such as ChatGPT, for mental health support in the past year, with 73.3% seeking help for anxiety, 63% personal advice, and 59.7% depression [1]. Furthermore, a recent Harvard Business Review report identified therapy and companionship as the leading AI use case in 2025, with finding purpose ranked third [2]. This analysis also highlights the shift in AI use for emotional support and personal development, as therapy and companionship rose from the second use case in 2024, and finding purpose was not identified in the top 100 use cases that year [2]. While recent studies from OpenAI and Anthropic report that smaller percentages of messages are exclusively for mental health support, they still imply hundreds of millions of mental health–related messages are being exchanged with ChatGPT and Claude each day [3,4].

Although AI tools are available 24/7 to provide support in a timely, accessible manner and initial research suggests that AI-based conversational agents may improve anxiety and depression symptoms [5], general AI tools are not designed using evidence-based practices or with the safety guardrails necessary for mental health use. Specifically, general LLMs may provide misinformation or hallucinate, perpetuate inequalities and stigma around mental health disparities, not store and protect personal data and information in accordance with HIPAA (Health Insurance Portability and Accountability Act) and standard medical regulations, provide inaccurate or inconsistent responses, not escalate care when a patient is indicating serious mental illness or self-harm, and fail to respond to crises [6,7]. Furthermore, general LLMs are designed to maximize engagement and create dependence [8], continuously asking the user more questions to keep them in the conversation, whereas mental health treatment is designed to provide patients with tools and skills that extend beyond care to promote personal growth, autonomy, and long-term well-being [9]. AI tools offer great promise for improving the accessibility of mental health support; however, it is imperative to design tools built specifically for mental health by experts that include informed consent, evidence-based practices, safety mechanisms, regulation compliance, and rigorous research and testing.

Headspace [10] is a commercial digital mental health platform that offers a spectrum of mental health care options including mindfulness, meditation, and sleep content; cognitive behavioral therapy-based clinical programs; human-delivered care including coaching, therapy, psychiatry; and Ebb [11], a unique offering within the Headspace app that is an empathetic conversational artificial intelligence tool (CAI tool) designed to process thoughts and emotions and guide members to relevant Headspace content. Specifically, the CAI tool was designed, built, and tested by clinical psychologists and research experts for subclinical guidance using motivational interviewing techniques and clinically backed safety mechanisms (eg, safety escalation, high acuity safety message flagging and review, and AI risk detection). At Ebb’s launch in October 2024, early pilot data suggested that Headspace members used the CAI tool as a self-reflection tool for emotional support, with top conversation topics including relationship and social support, work or career frustration, and sleep challenges. Between launch and July 2025, the CAI tool was consistently monitored and updated to improve safety, usability, and LLM accuracy. Additionally, since its launch, the CAI tool has become available for more members, and its LLM was expanded to include more conversational prompts, Headspace content recommendations, more detailed memory knowledge (to previous conversations with the CAI tool and overall Headspace content consumption), and a more robust approach to safety risk detection.

As AI technologies and their applications in mental health rapidly evolve, it is critical to understand what individuals expect from these tools, how they are using them in real-world settings, and how they envision such tools fitting into their overall mental health journey. Therefore, the purpose of this study was to (1) understand the overall sentiment toward AI and expectations for a mental health CAI tool, (2) descriptively evaluate real-world use of the CAI tool, and (3) understand how Headspace members perceive the CAI tool fitting into their broader mental health journey.

## Methods

### Overview

This study used three data sources: (1) member survey: cross-sectional, mixed methods survey to understand general sentiment toward AI and expectations for t a mental health CAI tool; (2) app engagement data: real-world engagement analysis using in-app Headspace data to understand how Headspace members are using the CAI tool; and (3) diary study: qualitative study to understand how Headspace members perceive the CAI tool fitting into their broader mental health journey. Details on each data source are outlined below and identified in Table 1. Results are reported in accordance with STROBE (Strengthening the Reporting of Observational Studies in Epidemiology) reporting guidelines (Multimedia Appendix 1).

**Table 1:** Data sources used in this study.

Sources	Research questions	Description	Sample	Date	Outcomes
Member survey	What is the overall sentiment toward AI^a^, and what do Headspace members want from a mental health CAI^b^ tool?	Cross-sectional survey sent via Qualtrics	482 Headspace members who sent at least 1 message to the CAI tool	April 2025	Demographics, general AI use, AI attitudes (AIAS-4^c^), and CAI tools’ role in their mental health journey
App engagement data	How are Headspace members using the CAI tool?	In-app engagement metrics collected via the Headspace app	393,969 Headspace members who used the CAI tool	October 2024 to October 2025 (focus on engagement from July 25, 2025, to October 1, 2025)	Number of users, sessions, messages sent, messages received, session rating, and conversation themes
Diary study	How do Headspace members perceive a CAI tool fitting into their broader mental health journey?	Qualitative diary study conducted via Dscout	15 Headspace members	October 2025	Themes and topics from qualitative data focused on how a CAI tool fits within a daily routine, Headspace CAI tool vs. general AI tools, and barriers to engagement

^a^AI: artificial intelligence.

^b^CAI: conversational artificial intelligence.

^c^AIAS-4: Artificial Intelligence Attitude Scale-4.

### Member Survey: What Is the General Sentiment Toward AI, and What do Headspace Members Want From a Mental Health CAI Tool?

#### Data Collection

A cross-sectional survey was conducted to understand Headspace members’ general sentiment toward AI and their expectations for a mental health CAI tool. Eligible participants were US-based Headspace members aged 18 years or older who sent at least 1 message to the CAI tool. The survey was sent in April 2025 via Qualtrics [12] to Headspace members’ email addresses collected during Headspace onboarding. Questions included demographics (age, gender, and race), general AI use, attitudes toward AI measured using the AI Attitude Scale [AIAS-4, 4 questions on a scale from 1-10 with a total score as a mean of the 4 items (range 1-10, higher scores indicating more positive attitudes toward AI use)] [13], and how members want a CAI tool to play a role in their mental health journey. The full member survey is reported in Multimedia Appendix 2.

#### Data Analysis

Survey responses are reported descriptively with demographics, general AI use, and CAI tool expectations reported as frequencies and percentages; means and standard deviations reporting AIAS-4 individual items and total; and qualitative analysis for open-ended questions on the CAI tool’s role.

### App Engagement Data: How Are Headspace Members Using the CAI Tool?

#### Data Collection

Headspace app engagement data were collected from members who used the CAI tool from October 1, 2024, to October 1, 2025.

#### Data Analysis

To evaluate detailed engagement, metrics were calculated for three groups: (1) CAI tool 1.0: members who used the CAI tool from October 1, 2024, to July 24, 2025; (2) CAI tool 2.0: members who used the CAI tool from July 25, 2025, to October 1, 2025 as the CAI tool became available for more members and its LLM was expanded to include more conversational prompts, Headspace content recommendations, detailed memory knowledge (to previous conversations with the CAI tool and overall Headspace content consumption), and a more robust approach to safety risk detection; and (3) CAI tool 2.0 retained users: a subset of CAI tool 2.0 members who returned to the CAI tool twice within a week at least once during the study period (between July 25, 2025, and October 1, 2025). Detailed differences between CAI tool 1.0 and CAI tool 2.0 can be found in Table 2. To characterize the overall engagement funnel in all 3 groups, frequencies were reported for the total number of members who have used the CAI tool, total number of sessions, and total number of messages (sent and received). Mean (SD) values, and ranges (minimum to maximum) were reported for the number of sessions and messages per user and number of messages (sent and received) per session. To assess retention, mean (SD) values and ranges were reported for average monthly active users (MAU), weekly active users (WAU), and daily active users (DAU), and frequencies and percentages were reported for users who return within 7 days and return within 30 days. Frequencies and percentages were reported for postconversation thumbs-up ratings. In CAI tool 2.0, users with >1 conversation with the CAI tool, key conversation topics were pulled from conversation histories, with frequencies and percentages indicating the top 15 conversation topics.

**Table 2:** Updates made to the Headspace conversational artificial intelligence tool, differentiating CAI tool 1.0 and CAI tool 2.0.

Key updates	CAI^a^ tool 1.0	CAI tool 2.0
Study dates	October 1, 2024, to July 24, 2025	July 25, 2025, to October 1, 2025
LLM^b^ infrastructure	Turn-based prompting (ie, the CAI tool is able to ask a question or reflect throughout the session)	General conversation prompt, allowing a more flexible conversation
Memory	Reference to previous conversations at the start of a new session	Comprehensive memory of all previous conversations and more recent content plays
Content recommendations	End of session button prompted the option for a content recommendation	Content recommendations are woven into the conversation based on the LLM’s decision-making
Safety	In-house proprietary safety system (safety escalation, high acuity safety message flagging and review, AI^c^ risk detection)	Expanded in-house safety system with clearer clinical boundaries, reduced out-of-scope and sycophantic behavior, improved de-identification, evaluation of AI-related risks (eg, parasocial relationships), and continued expansion of safety flagging with clinician oversight

^a^CAI: conversational artificial intelligence.

^b^LLM: large language model.

^c^AI: artificial intelligence.

### Diary Study: How do Headspace Members Perceive a CAI Tool Fitting Into Their Broader Mental Health Journey?

#### Data Collection

A qualitative diary study was conducted with 15 Headspace members to understand how members perceive the CAI tool fitting into their broader mental health journey. Participants engaged in a 7-day diary study conducted via Dscout [14], where they shared their baseline comfort and usage of AI tools, reflected on their CAI tool engagement, and provided in-the-moment insights into their experiences using the CAI tool. Data from the diary study’s baseline assessment were used for the present analyses. Participants were recruited through Dscout’s research platform using a screener that confirmed Headspace membership and active CAI tool use. Baseline questions included feedback prompts focused on understanding members’ baseline routines and initial perceptions of the CAI tool, including broad questions such as what a typical day looks like, what tools they use to manage stress, and their use of and attitudes toward general AI tools. CAI tool-specific questions included first impressions, how the CAI tool fits into their current routine, and how their use of the Headspace CAI tool differs from general AI tools. Data were collected using a mix of video, open-ended text, close-ended questions, and photo entries to capture in-the-moment reflections on their CAI tool engagement and how their use fits into their typical day. All data were captured within the Dscout platform and were reviewed by the Headspace research team to ensure data accuracy and participant compliance.

#### Data Analysis

Data were analyzed using a mixed-methods approach. Quantitatively, close-ended responses were transformed into categorical attributes to enable grouping and comparison of participant insights. Open-ended text and video transcriptions were also processed using LLM-assisted analysis to support theme identification. Qualitative data were analyzed using reflexive thematic analysis to identify patterns in how members perceive a CAI tool within their mental health journeys [15]. An inductive, data-driven approach was used, with codes generated from participants’ accounts and iteratively refined through repeated engagement with the data. Themes captured the primary topics participants discussed regarding their use of Headspace, including when and how they used the tool, perceived benefits, and areas of friction. Throughout the process, analytic decisions were documented through memoing and reflexive note-taking.”

### Ethical Considerations

Participation for all studies was entirely optional, and the studies operated under Headspace’s umbrella Institutional Review Board protocol, which covers research use of app data and associated research activities (Pro00078213). Member survey participants provided consent for their data to be used in this study as a part of the survey, members agreed to the use of their in-app data for research purposes when acknowledging the Headspace terms and conditions [16] and privacy policy [17], and diary study participants completed informed consent through Dscout when signing up for the service and prior to the start of the study. Participants for the diary study received US $60 in compensation upon completing all diary entries. All data were analyzed in aggregate form, and deidentification procedures were applied to ensure participant privacy and confidentiality in alignment with ethical standards for research involving human participants.

## Results

### Member Survey

#### Attitudes Toward AI

Overall, 482 Headspace members completed the member survey via Qualtrics and were included in these analyses (full survey results are reported in Table 3). The majority of survey respondents were women (321/475, 67.6%) and identified their race as White (414/475, 87.2%). Most respondents used AI tools outside of the Headspace CAI tool (272/475, 57.3%), with the top types of AI-powered tools reported as AI-generated content (246/304, 81.0%), virtual assistants (237/306, 77.7%), AI chatbots for customer service (202/304, 66.2%), and AI-powered recommendation systems (175/305, 57.4%). While the majority of respondents reported using general AI tools, only a small percentage (12.5%) reported using other AI tools for mental health support or self-reflection outside of the Headspace CAI tool. Most people agreed that they would use AI in the future, but overall attitudes toward AI (ie, perception of impact on life, work, and humanity) were neutral (AIAS-4 mean 5.7, SD 2.2, range 1-10).

**Table 3:** Member survey quantitative results highlighting member demographics, artificial intelligence (AI) use, and attitudes toward AI (AIAS-4^a^).

Characteristics			Values
**Age (years), n (%)**			
	18-24		9 (1.9)
	25-34		53 (11.1)
	35-44		101 (21.2)
	45-54		109 (22.9)
	55-64		108 (22.7)
	65-74		79 (16.6)
	75 and older		17 (3.6)
**Gender, n (%)**			
		Woman	321 (67.6)
		Man	144 (30.3)
		Nonbinary	5 (1.1)
		Prefer to self-describe	2 (0.4)
		Prefer not to answer	3 (0.6)
**Race and ethnicity (choose all that apply), n (%)**			
		White or Caucasian	414 (87.2)
		Hispanic or Latino	27 (5.7)
		Asian	17 (3.6)
		Black or African American	12 (2.5)
		Native Hawaiian or Pacific Islander	4 (0.8)
		Native American or Alaskan Native	3 (0.6)
		Middle Eastern or North African	3 (0.6)
		Other	5 (1.1)
		Prefer not to answer	11 (2.3)
**Outside of Ebb, have you ever used an AI-powered tool or service?, n (%)**			
		Yes	272 (57.3)
		No	169 (36.6)
		Maybe	34 (7.2)
**What types of AI-powered tools have you used? Select all that apply (n=272, those who responded yes to using AI tools), n (%)**			
		AI-generated content	247 (81.0)
		Virtual assistants	237 (77.7)
		AI chatbots for customer service	202 (66.2)
		AI-powered recommendation systems	175 (57.4)
		AI-powered mental health or wellness tools	175 (57.4)
		AI image or video generators	64 (21.0)
		Other	19 (6.2)
**Outside of Ebb, have you used any other AI tools for mental health support or self-reflection?, n (%)**			
		Yes	59 (12.5)
		No	414 (87.5)
**AIAS-4^b^ (range 1-10), mean (SD)**			
		I believe that AI will improve my life.	5.6 (2.4)
		I believe that AI will improve my work.	5.7 (2.7)
		I think I will use AI technology in the future.	7.2 (2.6)
		I think AI technology is positive for humanity.	5.1 (2.3)
		Total	5.1 (2.3)

^a^AIAS-4: Artificial Intelligence Attitude Scale-4.

#### Perceptions of a Mental Health CAI Tool

When asked what role members would want a CAI tool to play in their mental health journey, top responses included tool or assistant to be more efficient (n=147, 35%), guide to navigate to mental health resources (n=138, 33%), and a coach to help set and achieve goals (n=125, 30%). Qualitative themes, topics, and quotes from the open-ended questions are reported in Multimedia Appendix 3. Members used the CAI tool as an interactive self-reflection tool to vent and reflect, appreciating the feedback and reflection it provides. Specifically, 1 member indicated that, “I use [Ebb] as a form of journaling and as a way to cope with anxious overthinking.” While members use the CAI tool to guide them toward content within the Headspace app, they emphasized the need for more personalized content recommendations. Results suggest that members also use the CAI tool as an interim support between other forms of care, want it to be an adjunct to their therapy, and requested direct connection to human-delivered care within Headspace – with 1 member indicating that, “Ebb provides support for the tough moments between seeing my mental health providers.” Finally, members voiced their concerns about trusting AI for mental health care, emphasizing the need for transparency in data safety and ethics, structure around clinical guidelines, and for CAI tools to be a resource in addition to human-delivered mental health care, not as a replacement. Additionally, members were hesitant to anthropomorphize a CAI tool, wanting it to be clearly identified as an AI tool. Members specifically said, “I would like to know what the confidentiality and security level is there,” and, “Ebb is not a therapist or real human, but I appreciate Ebb listening and providing feedback and recommendations. Ebb is more of a guide who points me to the direction I want to go.”

### App Engagement Data

#### CAI Tool 1.0

**Figure 1:**
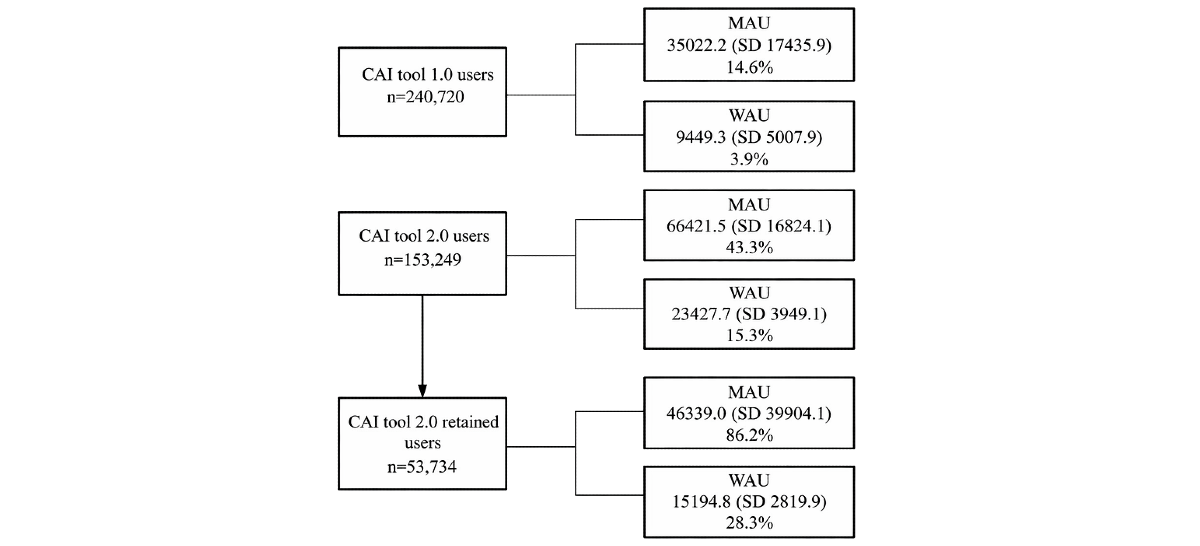
Engagement funnel from CAI tool 1.0 and CAI tool 2.0 (overall and retained users). CAI tool 1.0 and 2.0 users are mutually exclusive, with CAI tool 2.0 retained users being a subset of CAI tool 2.0. CAI: conversational artificial intelligence; MAU: monthly active user; WAU: weekly active user.

Real-world CAI tool use showed strong engagement across 393,969 unique members, with the overall CAI tool engagement funnel outlined in Figure 1 and the full app engagement data reported in Table 4. Overall, 240,720 Headspace members engaged with CAI Tool 1.0 (users from October 1, 2024, to July 24, 2025), exchanging 6,775,167 messages. Across the study period, 75,557 (25.8%) completed 2 sessions within 30 days, and 62,054 (25.8%) completed 2 sessions within 7 days. On average, 14.6% (mean 35,022.2, SD 17,435.9) are MAUs, and 3.9% (mean 9449.3, SD 5007.9) are WAUs, and members engaged in a total of 2.4 (SD 14.1) sessions, sent a total of 12.8 (SD 129.1) messages to the CAI tool, and received 15.3 (SD 168.3) messages from the CAI tool.

**Table 4:** In-app conversational artificial intelligence tool engagement data for Headspace members from October 1, 2024, to October 1, 2025.

Engagement	CAI tool 1.0 users^a^	CAI tool 2.0 users^b^	CAI tool 2.0 retained users^c^
Total number of users, N	240,720	153,249	53,734
Total sessions, N	568,611	436,104	326,178
Total messages sent, N	3,026,387	2,526,894	1,991,025
Total messages received, N	3,610,066	2,925,353	2,282,652
2 CAI^d^ tool sessions within 7 days, n (%)	68,701 (28.5)	77,894 (50.8)	53,734 (100)
2 CAI tool sessions within 30 days, n (%)	80,973 (33.6)	82,392 (53.8)	—^e^
Positive conversation rating (thumbs up)^f^, n (%)	94,308 (90.4)	37,819 (93.5)	46,935 (93.4)
Duration of session (minutes), mean (SD), range	6.7 (23.7), 0.1-4679.6	6.9 (12.6), 0.1-1173.6	7.6 (13.7), 0.1-1173.6
Monthly active users, mean (SD), range	35,022.2 (17435.9), 10,825-59,247	66,421.5 (16,824.1), 50,220-87,579	46,339.0 (39,904.1), 5860-88,062
Weekly active users, mean (SD), range	9449.3 (5007.9), 2433-17,640	23,427.7 (3949.1), 16,536-30,094	15,194.8 (2819.9), 10,879-20,389
Daily active users, mean (SD), range	1623.5 (950.9), 305-3867	4619.2 (963.5), 2373-7131	3432.8 (763.4), 1596-5152
Sessions per user, mean (SD), range	2.4 (5.9), 1-947	2.9 (9.1), 1-1107	6.1 (14.8), 1-1107
Messages sent per user, mean (SD), range	12.6 (44), 1-10,870	16.5 (75.8), 1-9183	37.1 (125.3), 2-9183
Messages received per user, mean (SD), range	15.0 (48.4), 0-11,569	19.1 (81.1), 0-9447	42.5 (133.7), 2-9447
Messages sent per session, mean (SD), range	5.3 (5.4), 1-274	5.8 (6.3), 1-460	6.1 (6.9), 1-460
Messages received per session, mean (SD), range	6.3 (5.6), 0-287	6.7 (6.4), 0-465	7 (7), 0-465

^a^Engagement from October 1, 2024, to July 24, 2025.

^b^Engagement from July 25, 2025, to October 1, 2025.

^c^Engagement from July 25, 2025, to October 1, 2025: a subset of conversational artificial intelligence tool 2.0 users who used the tool ≥2 days over a 7-day period.

^d^CAI: conversational artificial intelligence.

^e^Not available.

^f^Thumbs up rating was calculated based on those who rated their conversation with Ebb as a thumbs up or down (icons presented at the bottom of a conversation). Sample sizes are as follows: CAI tool 1.0 users n=106,008, CAI tool 2.0 users n=79,240, and CAI tool 2.0 retained users n=50,262.

#### CAI Tool 2.0

Since the launch of CAI tool 2.0 (July 25, 2025, to October 1, 2025), 153,249 members engaged with CAI tool 2.0, with 31,498 (20.6%) completing 2 sessions within 30 days and 53,734 (35.1%) completing 2 sessions within 7 days. On average, 43.3% (mean 66,421, SD 16,824.1) are MAUs and 15.3% (mean 23,427.7, SD 3,949.1) are WAUs. On average, members engaged in a total of 2.9 (SD 9.1) sessions, sent a total of 16.5 (SD 75.8) messages to the CAI tool, and received 19.1 (SD 81.1) messages from the CAI tool. Key conversation topics included Headspace app use and navigation, health and well-being, relationships, productivity, work and career, and anxiety and stress (Multimedia Appendix 4). Overall, 93.5% (37,819//40,449) of CAI tool users rated their conversation positively.

#### CAI Tool 2.0 Retained Users

**Figure 2:**
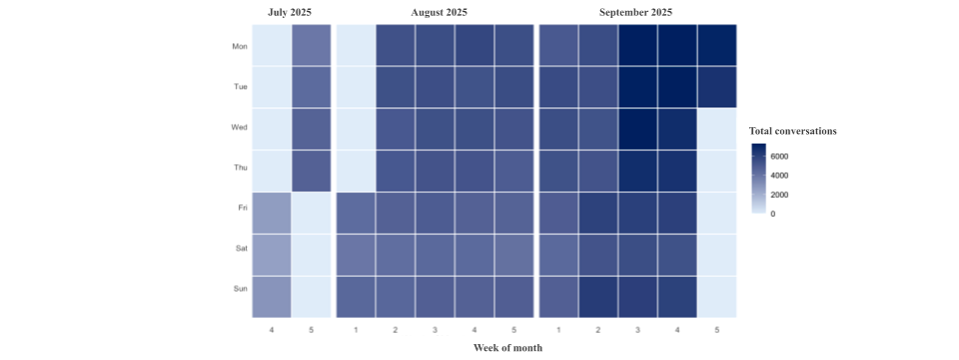
Total conversations across the study timeframe (July 25, 2025, to October 1, 2025) for CAI tool 2.0 retained users (2 sessions within 7 days).

In CAI tool 2.0, retained users (subset of CAI tool 2.0 members, those who engaged in at least 2 CAI tool sessions within 7 days anytime in the study period, n=53,734), on average, 86.2% are MAUs and 28.3% are WAUs. CAI tool 2.0 retained users engaged in a total of 326,178 sessions and exchanged 4,273,677 messages with the CAI tool, with 6.1 (SD 14.8) sessions per user, 37.1 (SD 125.8) messages sent per user, and 42.5 (SD 133.7) messages received per user. CAI tool 2.0 retained users who completed more conversations over time (Figure 2). Overall, 93.4% (46,935/50,250) of CAI tool retained users rated their conversation positively.

### Diary Study

Overall, 15 Headspace members completed the diary study baseline assessment, providing 1 Dscout entry each that combined open-ended reflections with several multiple-choice questions about their familiarity with AI tools, early impressions of the CAI tool, and anticipated use contexts. Overall, participants represented a highly engaged and technologically fluent sample; 73.3% (n=11) reported using AI tools such as ChatGPT (OpenAI), Google Gemini, or Copilot multiple times per day, and 13.3% reported using tools about once per day. Most (n=13, 86.7%) had already experimented with AI for health or wellness purposes, including stress management, exercise, or learning about symptoms, suggesting strong readiness to adopt CAI in a well-being context.

All participants were current Headspace users, and 73.3% (n=11/15) had used the CAI tool at least once. Overall, 66.7% (n=1015) described their first impression of the CAI tool as positive, with 46.7% (n=715) rating it somewhat positive and 20% very positive. A single participant expressed a somewhat negative view, with follow-up questions suggesting this participant is hesitant to share their feelings with AI in general. Participants generally found the CAI tool approachable and supportive, often describing it as a quick way to check in emotionally or regain focus, though several remained uncertain about how personalized or trustworthy its responses might be compared with other AI tools.

When asked when they imagined using the CAI tool throughout the week, participants most frequently selected moments of heightened emotion or daily transition. Overall, 80% (12/15) anticipated using the CAI tool when feeling anxious or overwhelmed, 60% (9/15) during stressful moments, and 46.7% (7/15) during morning routines, commutes, or while winding down at night. Overall, 33.3% (5/15) expected to use it during work or study breaks.

Open-ended responses and video transcriptions across the prompts reinforced the quantitative trends and revealed six inductive themes: (1) stress and anxiety coping, with members using the CAI tool to ground, reframe, and normalize emotions; (2) work/career focus, using the CAI tool to reset between meetings or during pressure; (3) interactive self-reflection, treating the CAI tool as a responsive diary; (4) sleep and nighttime routines, using the CAI tool to wind down and process the day; (5) content recommendations and navigation, valuing guidance to relevant meditations and courses; and (6) trust, privacy, and accuracy expectations, requesting transparency on data handling and more personalized guidance. Collectively, entries situate the CAI tool as an adjunct, between-session support that complements members’ broader mental-health toolkit rather than replacing human care. Members contrasted the CAI tool’s mental-health framing and content routing with general-purpose AI tools, and they emphasized opportunities to increase personalization and clarify privacy and safety. Key quotes for the open-ended response themes are reported in Multimedia Appendix 5.

## Discussion

### Principal Findings

The purpose of this study was to understand Headspace members’ overall sentiment toward AI and expectations for a mental health CAI tool, evaluate real-world use of the CAI tool, and understand how Headspace members perceive a CAI tool fitting into their broader mental health journey. Results from this study are imperative to the field of digital mental health, where AI is rapidly evolving, but limited insights exist on the details of real-world engagement and use cases. Despite reporting overall neutral attitudes toward AI, the majority of members reported using general AI tools. Members viewed the CAI tool as a valuable tool to process thoughts and reflect, connect to relevant Headspace content, and provide support in between other forms of care (eg, therapy). A substantial number of Headspace members interacted with the CAI tool during the study period, and iterative product improvements led to higher retention, greater engagement, and deeper conversations. Diary study results further highlight the CAI tool’s role as an accessible form of support that naturally integrates into moments of stress, transition, or reflection.

While our findings and broader AI engagement data indicate widespread adoption of generative AI tools [18], attitudes toward AI remain neutral to negative, and skepticism persists [19]. These results underscore the critical need for transparency in AI-driven mental health products. Although members expressed trust in Headspace, they emphasized the importance of understanding the confidentiality and security measures governing the data they share with the CAI tool. Members also requested that the CAI tool be clearly labeled as an AI tool to differentiate it from human-delivered care (eg, text-based mental health coaching or therapy). As the regulatory landscape evolves to catch up with AI advancements, it is important for companies developing mental health AI tools to enact minimum viable safeguards including: (1) transparent consumer labeling of intended use, benefits, and limitations; (2) safety by design principles to monitor for overuse, detect risk, and flag needs for escalation; and (3) child and adolescent safeguards to account for developmental differences in users. Beyond regulation, as AI adoption and attitudes continue to evolve, it will be essential to regularly assess how members perceive AI’s role in their mental health care experiences, particularly in relation to professional, human-delivered care.

In contrast to other mental health AI tools developed for therapeutic or clinical applications, the Headspace CAI tool was designed for subclinical support to help maintain mental wellness by fostering regular reflection and mindfulness. It is not a substitute for human-delivered care and does not provide clinical mental health services [11]. Member perceptions were consistent with the CAI tool’s intended subclinical use case, describing the CAI tool as a tool that promotes self-reflection, enables brief emotional resets and reflective pauses, guides them toward relevant Headspace mental health content and resources, and provides in-the-moment support between additional care, such as therapy sessions. Findings from the diary study further reinforce this view, with members seeing the CAI tool as a complementary aid that naturally fits within their daily routines and broader mental health ecosystem. Notably, members often turned to the CAI tool during moments of heightened emotion or during daily transitional periods (eg, commutes, work or study breaks, or while winding down at night). These patterns highlight an opportunity for mental health AI tools to engage people in real time during moments of activation—when stress, frustration, or sadness are high enough to spark openness to change, but not so high as to overwhelm [20]. While therapy and other traditional forms of support often focus on reflection after the fact, AI interventions like CAI tools can offer assistance in the moment, when the mind may be most receptive. Additionally, the CAI tool’s connection to Headspace’s extensive evidence-based content library allows for dynamic recommendation of the right mindfulness exercises, meditations, and/or cognitive-behavioral tools based on a member’s needs. Improving the CAI tool’s ability to more seamlessly navigate to licensed professionals when higher levels of support are needed, including coaches, therapists, and psychiatrists within the Headspace network, can ensure that members receive the right level of care at the right time. An integrated system of AI, content, and human care creates a safe, clinically informed continuum of support compared to stand-alone mental health AI tools.

The rapid evolution of general-purpose AI and related technologies creates new opportunities for the continued advancement of purpose-built mental health AI. In just over a year since launch, the CAI tool has undergone significant updates. Notably, its LLM was expanded to include more conversational prompts, Headspace content recommendations, enhanced memory capabilities (capturing prior conversations and Headspace content engagement), and a more robust approach to safety risk detection. These improvements aimed to increase personalization, strengthen safety mechanisms (eg, escalation pathways, high-acuity flagging, and AI-based risk detection), and foster deeper engagement with Headspace app content. Comparative engagement data between CAI tool 1.0 (preupdate) and CAI tool 2.0 (postupdate) reflect these enhancements. Descriptive results indicate higher retention, greater weekly activity, and more positive conversation ratings. Although the average number of sessions per user remained similar, the CAI tool 2.0 cohort exchanged more messages, suggesting increased conversational depth. Among retained CAI tool 2.0 users (those with 2+ sessions within 7 days), weekly retention continued to rise. Moreover, these retained users averaged more than twice as many sessions as those in the CAI tool 1.0 and the general CAI tool 2.0 cohort. Overall, this study’s engagement data suggests that enhanced memory, personalization, conversational depth, and tailored content recommendations contributed to stronger user retention, indicating that members are finding meaningful support and returning to the CAI tool more frequently. As users engage with AI more regularly, conversations likely become increasingly personalized, deepening user comfort and connection with the tool. Furthermore, as AI adoption and familiarity grow more broadly, individuals may become more open to engaging with mental health AI products, potentially leading to new patterns of real-world engagement over time.

### Strengths, Limitations, and Future Directions

A key strength of this study lies in its use of large-scale, real-world engagement data drawn from a widely used commercial mental health app. The multiple methods design integrating multiple data sources with both quantitative and qualitative as well as survey and real-world data, offers a nuanced understanding of how Headspace members engage with the CAI tool and provides valuable insight into how AI tools can be safely and effectively designed for mental health support.

While this study contributes important findings to the emerging field of AI in mental health, several limitations should be acknowledged. The data analyzed reflect Headspace members who used the CAI tool during its first year of availability, which may introduce selection bias toward individuals who are already comfortable using digital tools for mental health or who are early adopters of AI-based support. Additionally, data suggest that this study skewed toward a highly engaged and technologically fluent sample, which may limit generalizability. These analyses focus on descriptive surveys, engagement, and qualitative data. Although these descriptive findings help bridge an important knowledge gap in understanding real-world use of AI within digital mental health contexts, future research should aim to link engagement patterns with clinical outcomes to more fully assess the efficacy and impact of AI-driven mental health tools.

Headspace’s future product development for mental health CAI will focus on deeper integration across Headspace’s full spectrum of mental health resources, enabling members to use the CAI tool not only to access mindfulness and meditation content, but also to more seamlessly connect with licensed professionals. Additional advancements include the development of single-session interventions and conversation pathways designed to provide tailored, in-the-moment support. Continued development of all mental health AI tools should include transparent consumer labeling, safety by design principles, and child and adolescent safeguards in addition to continued advancements to expand memory, personalization, conversation depth, and coping skill development. Future research will expand beyond descriptive and feasibility studies to examine clinical efficacy and outcomes, exploring how the CAI’s ongoing evolution influences engagement, care adherence, clinical outcomes, and individual differences that may moderate these effects.

### Conclusions

AI tools offer tremendous promise for expanding access to mental health care; however, it is essential that such tools are purpose-built for mental health by experts with minimum viable safeguards including (1) transparent consumer labeling of intended use, benefits, and limitations; (2) safety by design principles to monitor for overuse, detect risk, and flag needs for escalation; and (3) child and adolescent safeguards to account for developmental differences in users. Ebb, Headspace’s empathetic CAI tool, was developed by clinical psychologists using motivational interviewing techniques and clinically informed safety systems to help members process thoughts and emotions while guiding them toward relevant content within the Headspace app. Findings from this study reveal that, despite generally neutral to negative attitudes toward AI, members are using AI tools and plan to continue use. Members viewed the CAI tool as a complementary mental health resource, fitting naturally into their broader system of care by connecting them to helpful content within the app and supporting reflection. Engagement patterns have evolved over time, with product updates leading to higher retention, greater engagement, and deeper conversations. Future development will focus on further integration with Headspace’s full spectrum of mental health services, while upcoming research will evaluate the CAI tool’s impact on clinical outcomes and care adherence. Continued, clinically informed innovation in AI for mental health is essential to ensure that these tools remain safe, effective, and supportive for individuals seeking accessible mental health care.

## Appendix

Multimedia Appendix 1 STROBE checklist.

Multimedia Appendix 2 Member survey questions to understand overall sentiment toward AI and what do Headspace members want from a mental health AI tool.

Multimedia Appendix 3 Member survey qualitative results highlighting themes, topics, and key quotes from open-ended survey questions.

Multimedia Appendix 4 CAI tool 2.0 conversation topics synthesized via in app data collection.

Multimedia Appendix 5 Key themes and CAI tool use cases from the diary study open-ended questions.

## Abbreviations

AI: artificial intelligence
AIAS-4: Artificial Intelligence Attitude Scale-4
CAI: conversational artificial intelligence
DAU: daily active user
HIPAA: Health Insurance Portability and Accountability Act
LLM: large language model
MAU: monthly active user
STROBE: Strengthening the Reporting of Observational Studies in Epidemiology
WAU: weekly active user
