# Supplementary material for: Real-World Use of a Mental Health AI Companion: Multiple Methods Study
Source: JMIR Form Res. 2026 Feb 13;10:e86904. doi: 10.2196/86904 (PMC12949398; doi:10.2196/86904)
Supplement: Multimedia Appendix 1 [file formative_v10i1e86904_app1.pdf]

**Supplemental 1. STROBE checklist.**	
**Recommendation**	**Checklist**
**Title and abstract**	
Indicate the study's design with a commonly used term in the title or the abstract	X
Provide in the abstract an informative and balanced summary of what was done and what was found	X
**Introduction**	
Background and Rationale	
Explain the scientific background and rationale for the investigation being reported	X
Objectives	
State specific objectives, including any prespecified hypotheses	X
**Methods**	
Study design	
Present key elements of study design early in the paper	X
Setting	
Describe the setting, locations, and relevant dates, including periods of recruitment, exposure, follow-up, and data collection	X
Participants	
Give the eligibility criteria, and the sources and methods of selection of participants	X
Variables	
Clearly define all outcomes, exposures, predictors, potential confounders, and effect modifiers. Give diagnostic criteria, if applicable	X
Data sources/ measurement	
For each variable of interest, give sources of data and details of methods of assessment (measurement). Describe comparability of assessment methods if there is more than one group	X
Bias	
Describe any efforts to address potential sources of bias	X
Study size	
Explain how the study size was arrived at	X
Quantitative variables	
Explain how quantitative variables were handled in the analyses. If applicable, describe which groupings were chosen and why	X

Statistical methods	
(a) Describe all statistical methods, including those used to control for confounding	X
(b) Describe any methods used to examine subgroups and interactions	X
(c) Explain how missing data was addressed	X
**Results**	
Participants	
(a) Report numbers of individuals at each stage of study—eg numbers potentially eligible, examined for eligibility, confirmed eligible, included in the study, completing follow-up, and analysed	X
(b) Give reasons for non-participation at each stage	X
(c) Consider use of a flow diagram	X
Descriptive data	
(a) Give characteristics of study participants (eg demographic, clinical, social) and information on exposures and potential confounders	X
(b) Indicate number of participants with missing data for each variable of interest	X
Outcome data	
Report numbers of outcome events or summary measures	X
Main results	
(a) Give unadjusted estimates and, if applicable, confounder-adjusted estimates and their precision (eg, 95% confidence interval). Make clear which confounders were adjusted for and why they were included	X
(b) Report category boundaries when continuous variables were categorized	X
Other analyses	
Report other analyses done—eg analyses of subgroups and interactions, and sensitivity analyses	X
**Discussion**	
Key results	
Summarise key results with reference to study objectives	X
Limitations	
Discuss limitations of the study, taking into account sources of potential bias or imprecision. Discuss both direction and magnitude of any potential bias	X
Interpretation	
Give a cautious overall interpretation of results considering objectives, limitations, multiplicity of analyses, results from similar studies, and other relevant evidence	X

Generalisability	
Discuss the generalisability (external validity) of the study results	X
**Other information**	
Funding	
Give the source of funding and the role of the funders for the present study and, if applicable, for the original study on which the present article is based	X
