# Supplementary material for: Real-World Use of a Mental Health AI Companion: Multiple Methods Study
Source: JMIR Form Res. 2026 Feb 13;10:e86904. doi: 10.2196/86904 (PMC12949398; doi:10.2196/86904)
Supplement: Multimedia Appendix 2 [file formative_v10i1e86904_app2.pdf]

Supplemental 2. Member survey questions to understand overall sentiment toward AI and what do Headspace members want from a mental health AI tool.

Demographics

- How old are you?
  - <18
  - 18-24
  - 25-34
  - 35-44
  - 45-54
  - 55-64
  - 65-74
  - 5+
- What gender do you most identify with
  - Woman
  - Man
  - Non-binary
  - Prefer not to answer
  - Prefer to self-describe (please specify)
- How do you identify your race or ethnicity? Select all that apply.
  - Asian
  - Native Hawaiian/Pacific Islander
  - Native American/Alaskan Native
  - Black/African American
  - Hispanic/Latino/Latine/Spanish
  - White/Caucasian
  - Middle Eastern/North African
  - Prefer not to answer
  - Other (please specify)

AI Use History

- Outside of Ebb, have you ever used an AI-powered tool or service?
  - Yes
  - No
  - Maybe
- Which types of AI-powered tools have you used? Select all that apply.
  - Virtual assistants (e.g., Siri, Alexa, Google Assistant)
  - AI chatbots for customer service
  - AI-powered mental health/wellness tools (e.g., Woebot, Replika, Ebb)
  - AI-generated content (e.g., ChatGPT, Gemini, Copilot)
  - AI image/video generators (e.g., DALL-E, Midjourney, Runway)
  - AI-powered recommendation systems (e.g., Netflix, Spotify, Youtube suggestions)
  - Other (please specify)
- How often do you use AI-powered tools?
  - Daily

    - A few times a week
    - A few times a month
    - Rarely
    - Never
- Outside of Ebb, have you used any other AI tools for mental health support or self-reflection?
    - Yes
    - No
- Please indicate what AI tools you have used for mental health support or self-reflection.

AIAS-4
The following questions are about your attitudes towards artificial intelligence (AI). Rate from 1(not at all) to 10 (completely agree).
- I believe that AI will improve my life.
- I believe that AI will improve my work.
- I think I will use AI technology in the future.
- I think AI technology is positive for humanity.

Headspace Specific Questions
- As you reflect on your experience with Ebb so far and the role that it plays in your life, what do you currently see Ebb as? Ebb feels like a…
    - Friend/companion who is an empathetic listening ear, always by my side
    - Coach who helps me set and achieve my goals and fuels my personal growth
    - Therapist who helps me better understand my past and address my current mental health challenges
    - Tutor/teacher who helps me learn new skills
    - Guide who helps me navigate to the most helpful mental health resources
    - Tool/assistant who helps me be more effective or efficient
    - Other
- Please share the reasoning behind your previous response.
- What role would you like Ebb to play in your life in the future? In the future I would like Ebb to feel like a...
    - Friend/companion who is an empathetic listening ear, always by my side
    - Coach who helps me set and achieve my goals and fuels my personal growth
    - Therapist who helps me better understand my past and address my current mental health challenges
    - Tutor/teacher who helps me learn new skills
    - Guide who helps me navigate to the most helpful mental health resources
    - Tool/assistant who helps me be more effective or efficient
    - Other
- Please share the reasoning behind your previous response.
- Which features would you most like to see added to Ebb? Please drag and rank the following options from most (1) to least (12) preferred. I would like Ebb to...
    - Help me find content in the Headspace app
    - Integrate with Headspace care services (coaching, therapy)
    - Guide self-reflection exercises and provide journaling prompts

    - Help me stay consistent (i.e., be an accountability partner for Headspace courses and programs)
    - Personalize mindfulness and meditation content recommendations
    - Ask daily mental health check-ins and mental health symptom surveys
    - Help me set and achieve my goals
    - Support me through relationship navigation and communication support
    - Send motivational messages and affirmations
    - Provide educational content on mental health topics
    - Interact and speak with me via voice in addition to text
    - Provide in the moment support for more urgent needs
- Do you have any additional thoughts to share on your experience with Ebb or other AI tools?
