# Supplementary material for: Real-World Use of a Mental Health AI Companion: Multiple Methods Study
Source: JMIR Form Res. 2026 Feb 13;10:e86904. doi: 10.2196/86904 (PMC12949398; doi:10.2196/86904)
Supplement: Multimedia Appendix 3 [file formative_v10i1e86904_app3.pdf]

**Supplemental 3. Member survey qualitative results highlighting themes, topics, and key quotes from open ended-survey questions.**		
**Themes**	**Topics**	**Quotes**
Journaling tool	Interactive journaling<br><br>Venting about life stressors<br><br>Self-reflection and processing	“I use it as a form of journaling and as a way to cope with anxious overthinking”<br><br>“I just use Ebb to vent when overwhelmed”<br><br>“To reflect, to say it out loud and get some feedback”<br><br>“Tool to work through an issue or thought pattern”
Headspace guidance	Headspace app guidance and navigation<br><br>Receiving personalized content recommendations	“I use Ebb to guide me to a potential meditation, podcast episode, etc on the Headspace app for relief”<br><br>“Ebb listens to me and recommends meditations”<br><br>“Learn more about the Headspace app as well as mindfulness in general"
In the moment support and accessibility	Interim support between care appointments<br><br>In the moment emotional regulation<br><br>24/7 availability<br><br>Adjunct to therapy / other mental health care	“I use Ebb when I am feeling overwhelmed by anxiety or depression and need immediate support”<br><br>“Ebb provides support for the tough moments between seeing my mental health providers”<br><br>“It provides perspective and support in the moment”<br><br>“Not a full substitute for human-to-human counseling, but an additional resource”<br><br>“Ebb is an adjunct to my human caregivers”

AI trust and skepticism	Safety concerns<br><br>Ethical implications<br><br>Differentiating AI mental health tools vs. generic tools	“I would not have tried it except for my trust in Headspace. And likely will not use any other AI technology”<br><br>“I would like to know what the confidentiality and security level is there”
**Note:** Themes, topics, and quotes were pulled from the following open-ended survey questions: 1) How do you use Ebb in addition to your mental health care (coaching, therapy, etc.; provided by or outside of Headspace) to support your mental health?; 2) As you reflect on your experience with Ebb so far and the role that it plays in your life, what do you currently see Ebb as?; 3) What role would you like Ebb to play in your life in the future?; and 4) Do you have any additional thoughts to share on your experience with Ebb or other AI tools?		
