# Supplementary material for: Real-World Use of a Mental Health AI Companion: Multiple Methods Study
Source: JMIR Form Res. 2026 Feb 13;10:e86904. doi: 10.2196/86904 (PMC12949398; doi:10.2196/86904)
Supplement: Multimedia Appendix 4 [file formative_v10i1e86904_app4.pdf]

**Supplemental 4. CAI tool 2.0 conversation topics synthesized via in app data collection.**		
**Topic Category**	**Keywords**	**Number of Messages**
Headspace app usage	Ebb, Headspace, app, first session, get started, learn more	652,516
Health and wellbeing	Health, pain, medical, cancer, sick, doctor, surgery, diagnosis, illness, injury, ocd, adhd, depression, trauma, bipolar, ptsd, healing, medication, therapy, recovery, COVID	8,344
Relationships	Marriage, connection, relationship, family, friend, partner, dating, husband, wife, mom, dad, son, daughter, sister, brother, roommate, caregiver, in laws	7,220
Productivity work/career	Work, job, career, shifts, boss, colleague, office, meeting, professional, coworker, manager, procrastination, motivation, focus, distracted, time management	6,328
Anxiety/stress	Anxiety, dress, overwhelmed, burnout, worry, frustrated, panic, pressure, nervous, tense, fear, scared, guilt	5,959
Sleep	Sleep, wake up, insomnia, awake, dream, bedtime, tired, exhausted, fatigued, nightmares, middle of the night	4,020
Self-image/acceptance	Self-acceptance, body, self-esteem, confidence, self-love, self-worth, self-care, self-compassion, self-criticism, body image, perfectionism, unattractive, unappreciated, self-talk	2,563
Grief/loss	Grief, grieving, loss, death, passed away, funeral, missing someone, miscarriage, divorce, end of a relationship/friendship, mourning	2,194
Hobbies/leisure	Companion, pet, games, golf, soccer, athletics, holiday, trip, adventure, music, creative, hobbies, leisure, gaming, cinema, gardening, carpentry, sewing	1,768
Finances/life decisions	Financial, money, debt, bills, moving, decision, choice, stretching finances, loans	1,520
Sexuality/identity	Sexuality, sexual identity, gender, gay, lesbian, bi	1,309
Mindfulness practice	Meditation, mindfulness, breathing exercises, daily meditation, evening meditation, guided meditation, recommendations	1,045

Productivity goal setting	Habit, routine, procrastination, motivation, purpose, goals, focus, distracted	986
Crisis and safety[a]	Call 988, text 988, lifeline, suicidal	838
Sadness and loneliness	Sadness, sad, loneliness, low, empty, isolated, depressed, hopeless	698
**Note:** Messages are able to be categorized in more than one topic category.<br>[a]All messages that fall within the *crisis and safety* category are flagged by our in-house proprietary safety/risk detection system and reviewed by clinicians.		
