# Supplementary material for: Real-World Use of a Mental Health AI Companion: Multiple Methods Study
Source: JMIR Form Res. 2026 Feb 13;10:e86904. doi: 10.2196/86904 (PMC12949398; doi:10.2196/86904)
Supplement: Multimedia Appendix 5 [file formative_v10i1e86904_app5.pdf]

**Supplemental 5. Key themes and CAI tool use cases from the diary study open ended questions.**		
**Theme**	**Ebb Use**	**Quotes**
Stress and anxiety coping	Ground, reframe, and normalize emotions	"I use it for a quick hit to feel more grounded or to remind me that a feeling will pass."<br><br>"I've used Ebb at points when I've been particularly anxious and need to have an empathetic conversation to work through thoughts without feeling judged."
Work/career focus	Reset between meetings or during pressure	"It is something that has definitely helped me in the workday, plus my day in general. It's a great way to center yourself and destress."<br><br>"I have had the app suggest that I use Ebb, but it's advertised as like an AI therapist and I don't feel like I need that during the day."
Interactive self-reflection	Responsive diary	"I use it like a journal that actually talks back and helps me process what's going on."<br><br>"I've been trying out Ebb with navigating things I'm experiencing in the moment when I don't want to sound crazy by crashing out to another person."
Sleep and nighttime routines	Wind down and process the day	"I reach for Ebb at night when my thoughts keep me up."<br><br>"I often use Ebb when I am experiencing emotions I want to talk about but they may feel too heavy for me to talk about with someone else."
Content recommendations and navigation	Guidance to relevant meditations/courses	"I have used Ebb in the past to help me find meditations when I am feeling a specific emotion. I can see myself using Ebb for that again."

**Supplemental 5. Key themes and CAI tool use cases from the diary study open ended questions.**		
		“Ebb helps me to stay grounded and motivated. I feel like I get quality information and ideas from Ebb. It helps me with my daily routine.”
Trust, privacy, and accuracy expectations	Requesting transparency on data handling and more personalized guidance	“I want to understand what happens with what I share.”<br><br>“I don’t really use it because it feels fake to me. I’m not sure—I don’t really feel like for me it serves a purpose.”<br><br>“I have used Ebb but I have trouble sort of fitting it into my regular routine. I’ve asked questions about the app and where the information goes.”
